# EVALUATING NURSING HOME-BASED INTERVENTIONS ON COMPETENCY LEVELS

**DOI:** 10.1093/geroni/igae098.1375

**Published:** 2024-12-31

**Authors:** Amy Recker, Ellen McCreedy, Rosa Baier, Kate Smith, Regina George, Sophia Geisser, Vanessa Aguilar, A Lynn Snow

**Affiliations:** Brown University, Providence, Rhode Island, United States; Veterans’ Administration, Providence, Rhode Island, United States; Brown University, Providence, Rhode Island, United States; University of Alabama, Tuscaloosa, Alabama, United States; University of Alabama, Tuscaloosa, Alabama, United States; University of Alabama, Tuscaloosa, Alabama, United States; University of Alabama, Tuscaloosa, Alabama, United States; University of Alabama, Tuscaloosa, Alabama, United States

## Abstract

The intervention at the heart of the 40WINKS sleep trial relies on a principle that can be applied to many other nursing home-based interventions: Progress through the intervention requires a gradual and consistent accumulation of skills that build upon one another. Without completing a fundamental level of the intervention (e.g., begin to hold staff huddles regularly), a nursing home’s team could not incorporate advanced concepts (e.g., engage in collaborative problem solving during huddles). With this in mind, the 40WINKS research team decided to evaluate the participating nursing homes based on progressive levels of competency. We evaluated levels of competency for each of the seven core elements of the intervention (leadership team functioning; huddles; problem solving; small action plans; individualized care; continuous metacognition; sleep and daytime activity information; connecting day and night staff; and spillover, spread, and integration), scoring each core element as not met, foundational, proficient, or advanced. We developed a matrix with the seven core elements and examples of the corresponding competency levels, which research team members complete weekly. We aggregate the competency matrices on a monthly basis and meet to code each nursing home into one of the four competency levels overall. This presentation will delve more in depth into this process, provide a roadmap for applying this approach to other interventions, and share preliminary results.

